# Towards the activity of twisted acyclic amides†

**DOI:** 10.1039/d5ra00229j

**Published:** 2025-03-24

**Authors:** Michele Tomasini, Lucia Caporaso, Michal Szostak, Albert Poater

**Affiliations:** a Institut de Química Computacional i Catàlisi, Departament de Química, Universitat de Girona C/Maria Aurèlia Capmany 69 17003 Girona Catalonia Spain albert.poater@udg.edu; b Dipartimento di Chimica e Biologia, Università di Salerno Via Ponte don Melillo 84084 Fisciano Italy; c Department of Chemistry, Rutgers University 73 Warren Street Newark NJ 07102 USA ms2223@newark.rutgers.edu

## Abstract

*N*,*N*-Boc_2_ amides have emerged as the most common class of acyclic twisted amides that have been engaged in a range of C–N activation and cross-coupling processes of ubiquitous amide bonds. These amides are readily synthesized from primary amides through a site-selective *tert*-butoxycarbonylation. Due to the steric bulk of di-*tert*-butoxy groups, these amides exhibit significant C=N bond twisting, which promotes N–C bond cleavage, facilitating their use in cross-coupling reactions. Herein, we present a computational blueprint for the C=N bond rotation in *N*,*N*-Boc_2_ amides, revealing that the rotational barrier and twist angle (*τ*) are influenced by the nature of the substituents at the sp^2^ carbon position. Sterically hindered substituents exhibit the highest distortions, leading to lower rotation barriers. Rotation along the C=N bond is accompanied by phenyl ring rotation to minimize steric clashes. A strong correlation between the rotational barriers and the HOMO energies is observed. These findings provide key insights into the fundamental role of amide bond distortion in C–N activation processes.

## Abstract 2

*N*,*N*-Boc_2_ amides have emerged as the most common class of acyclic twisted amides that have been engaged in a range of C–N activation and cross-coupling processes of ubiquitous amide bonds.

## Graphical abstract

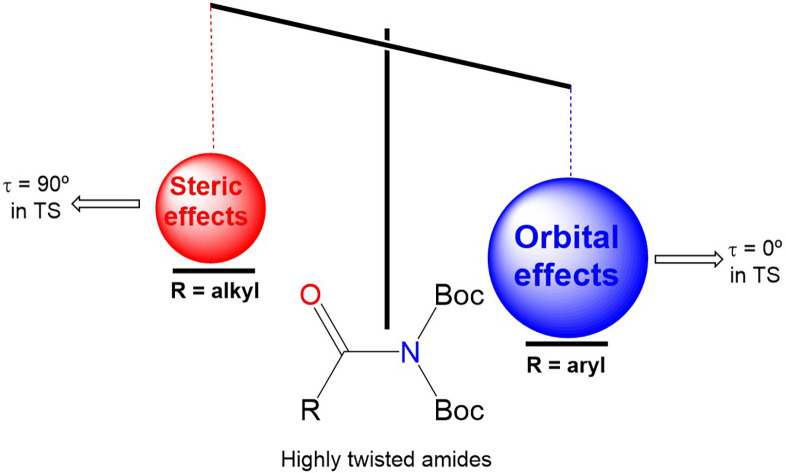

## Introduction

The amide bond is one of the most significant functional groups in both chemistry and biology.^1,2^ Historically, the Pauling resonance theory^3^ predicted that amides are predominantly planar structures, as confirmed by Density Functional Theory (DFT) calculations,^4^ and it has been extensively studied in chemistry, biochemistry, structural biology and materials science. The reason is that the planar nature of amides significantly influences the chemical reactivity, structural stability, and their properties. For example, inactivated amides exhibit an extremely slow rate of neutral hydrolysis, with a half-life of approximately 500 years,^5^ and the planarity of amide bonds plays a pivotal role in the formation of secondary structures like the α-helix in proteins. The chemical properties of amides, such as their preference for *O*-protonation, are also tightly linked to their planar geometry.^6^

Numerous studies have demonstrated that any deviation from planarity in amides dramatically alters their stability and reactivity.^7^ Amide bond twisting,^8,9^ in particular, has been shown to play a key role in various biological and chemical processes. For instance, it is central to enzymatic reactions such as *cis*–*trans* isomerization,^10,11^ amide hydrolysis,^12^ and protein splicing.^13^ More recently, it has been suggested that amide bond twisting is involved in protein *N*-glycosylation mechanisms and is a crucial factor in selective amide bond activation for cross-coupling reactions.^14^ This highlights the potential of exploiting amide bond twists as a valuable tool in modern synthetic organic chemistry.^15–17^

The most common approach for inducing significant twisting in amide bonds involves embedding the amide group into rigid cyclic ring systems.^18^ These bicyclic bridgehead lactams possess extreme geometric distortions,^19^ with Winkler–Dunitz parameters showing amide bond twisting angles (*τ*) as high as 90° and nitrogen pyramidalization (*χ*_N_) values up to 60°.^20^ However, synthesizing these highly twisted amides is notoriously difficult due to the substantial reduction in amide resonance stabilization,^21^ as demonstrated by the challenges in synthesizing 2-quinuclidonium tetrafluoroborate and 1-aza-2-adamantanone.^22,23^ In fact, these latter ones often involve complex and non-trivial transformations.

In recent years, researchers have explored alternative strategies to induce amide bond twisting. In particular, Kumagai, Shibasaki, and colleagues introduced a novel class of nonplanar amides with predominantly pyramidalized amide bonds achieved through peripheral coordination of Pd(ii) to nitrogen-based Lewis bases.^24^ This method generated amides with significant distortions from planarity, exhibiting *χ*_N_ values of up to 56° and *τ* of 19°. However, the need for metal coordination adds complexity to the distortion of common acyclic amides. Other approaches, such as the development of *N*-tetramethylpiperidine (TMP),^25^*N-*glutarimide,^26^ and *N*-1,3-thiazolidine-2-thione amides,^27^ have provided access to nonplanar amide geometries. Unfortunately, these methods often involve derivatives from carboxylic acids and suffer from issues like high hydrolysis susceptibility, as seen with TMP amides.

In 2018, Szostak and coworkers reported the synthesis and structural analysis of *N*,*N*-di-Boc amides, prepared directly from common benzamides *via* selective *N*,*N*-di-*tert*-butoxycarbonylation under mild conditions.^28^ This one-step process induces significant twisting of the amide bond, which is directly applicable to primary amides^29^ that are prevalent in organic synthesis and pharmaceutical industry.^30^ At present, *N*,*N*-Boc_2_ amides have been established as the most common class of acyclic twisted amides that have been engaged in a range of C–N activation and cross-coupling processes of ubiquitous amide bonds.^14–17^ From a structural standpoint, *N*,*N*-Boc_2_ amides serve as models for exploring amide bond distortion, offering a straightforward approach to induce non-planarity.^28–31^ The di-Boc strategy effectively distorts the primary amide bond, offering valuable insights into the geometry and reactivity of twisted amides,^31^ with potential applications in organic and pharmaceutical chemistry^32^ as well as in biochemistry, organic synthesis, and the design of molecular switches.^33^

In this manuscript, we present a computational blueprint for the C=N bond rotation in *N*,*N*-Boc_2_ amides, at the at the B3LYP-D3/6-311++G(d,p)(SMD(toluene))//B3LYP-D3/6-311++G(d,p) level of theory. These findings provide key insights into the fundamental role of amide bond distortion in C–N activation processes. The study introduces a novel approach to achieve significant amide bond twisting in acyclic amides *via* simple di-*tert*-butoxycarbonylation. This method allows for the controlled and reversible distortion of amide bonds, opening up new predictive avenues,^34,35^ for the study and application of nonplanar amides in various disciplines. In particular, our research offers a new approach to inducing nonplanarity in amides without relying on complex ring systems, positioning these amides as acyclic twisted structures.

## Computational details

Computational details: All DFT calculations were carried out with the Gaussian 16 set of programs.^36^ The electronic configuration of the molecular systems was depicted using the hybrid GGA functional developed by Becke, Lee, Parr and Yang (B3LYP),^37,38^ using the Ahlrichs basis set 6-311++G(d,p).^39^ As corrections stemming from dispersion play a crucial role in studying reactivity, we have incorporated them using Grimme's GD3 method.^40^ Geometry optimizations were conducted without symmetry constraints, and the characterization of the stationary points was accomplished through analytical frequency calculations. These frequencies were employed for computing unscaled zero-point energies (ZPEs), thermal corrections, and entropy effects at 298.15 K and 1 atm. Solvent effects were estimated with the universal solvation model SMD from Cramer and Truhlar using toluene as solvent.^41,42^ The reported Gibbs energies were obtained at the B3LYP-D3/6-311++G(d,p)(SMD(toluene))//B3LYP-D3/6-311++G(d,p) level of theory. In addition, to point out that scans of any group that could rotate were performed. Moreover, we performed a total of 1 ns of MD simulations with Langevin thermostat at 1000 K for each compound by using the Atomic Simulation Environment (ASE) and the PreFerred Potential (PFP).^43^ A total of 100 snapshots per simulation were minimized following the same minimization protocol described previously. The lowest energy-minimized snap was used for quantum mechanics calculations.

## Results and discussion

Starting from acyclic *N*,*N*-Boc_2_ amides, the steric hindrance of di-*tert*-butoxy groups was studied as shown in Table 1. In detail, these amides exhibit significant twisting of the C=N bond, promoting N–C bond cleavage. The computational analysis is based on the C=N bond rotation for a series of amides in this class.^44^ The entries differ in their substituents at the sp^2^ carbon position, and with the exception of sterically hindered groups, particularly ^*t*^Bu in entry 4, they exhibit a significant twist angle (*τ*). The amide with a *tert*-butyl group (entry 4) shows a large distortion, with *τ* values of 73.5°, to avoid steric clashes between the *tert*-butyl groups. Significant distortions (*τ* > 30°) are also observed in entries 6–13, which have different substituents in the *para* position of the phenyl ring.

**Table 1:** Rotational barrier of amide bond for different *N*,*N*-Boc_2_ amides (in kcal mol^−1^) and relative Winkler–Dunitz parameters and % *V*_Bur_

Entry	R	Δ*G*^‡^	*τ*	*χ*_N_	Σ*τ* + *χ*_N_	% *V*_Bur_
1	H	8.5	1.9	1.4	3.3	59.3
2	Me	3.8	2.5	2.0	4.5	67.3
3	^i^Pr	2.7	4.6	1.3	3.4	75.8
4	^*t*^Bu	0.5	73.5	10.7	84.2	82.8
5	CF_3_	3.1	28.9	14.4	43.2	76.9
6	Ph	4.4	40.8	13.7	54.5	81.8
7	4-NMe_2_Ph	9.1	51.3	11.4	62.7	81.7
8	4-OMePh	5.8	45.5	13.2	58.7	81.6
9	4-FPh	4.7	41.6	14.0	55.6	81.5
10	4-ClPh	4.3	40.0	14.1	54.1	81.7
11	4-CF_3_Ph	3.5	36.8	14.3	51.1	81.7
12	4-CNPh	3.6	36.9	14.4	51.3	81.6
13	4-NO_2_Ph	3.1	35.0	14.3	49.3	81.6

As reported previously, these amides are highly destabilized, with Winkler–Dunitz parameters (*τ*) ranging from 35.0° to 51.3°. The twist angle (*τ*) is influenced primarily by electron-donating groups (EDGs), with entries 7 and 8 showing more twisting than the unsubstituted amide (entry 6). In contrast, the presence of halides induces a distortion similar to that in entry 6, while purely electron-withdrawing groups (EWGs), as in entries 11–13, reduce the *τ* value. Finally, the different substituents on the phenyl ring do not significantly affect the nitrogen hybridization (*χ*_N_), which is generally more influenced by bulky groups attached to the carbonyl group of the amide bond.

**Fig. 1:**
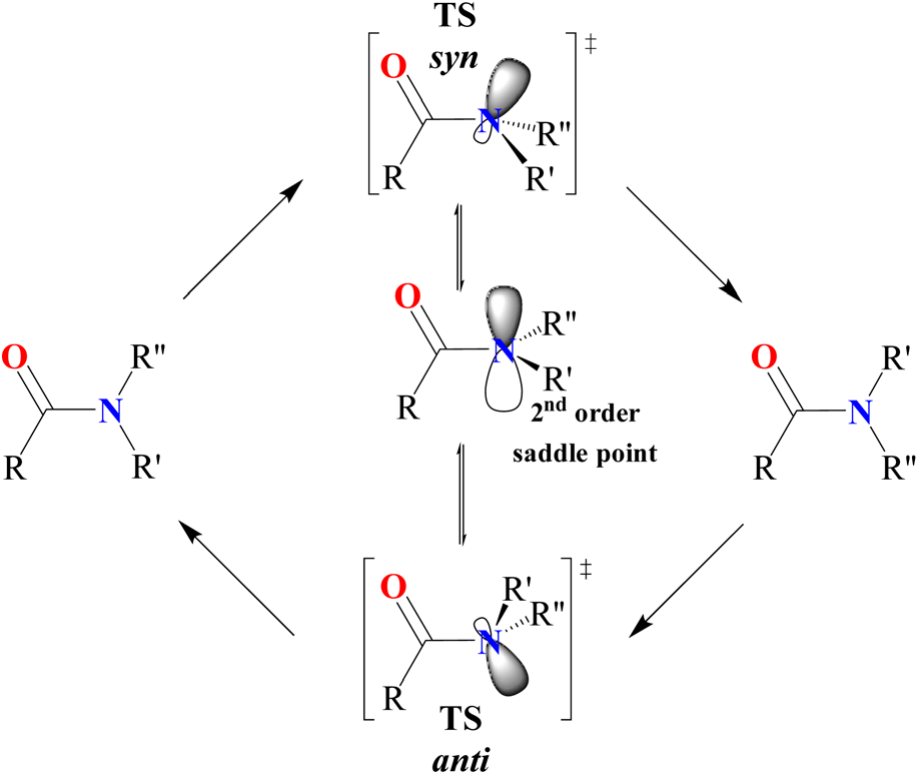
Classical mechanism of *cis–trans* isomerization for amides.

A detailed analysis of C=N bond rotation was conducted for each entry in Table 1, with the resulting activation energy barriers ranging from 0.0 to 10.9 kcal mol^−1^. As reported in the literature, amide bond rotation occurs through two possible transition states: TS-*anti* and TS-*syn*,^45–47^ where the nitrogen adopts sp^3^ hybridization. As illustrated in Fig. 1, these two transition states differ based on the position—*anti* or *syn*—of the lone pair orbital lobe relative to the C=O bond. The TS-*anti* and TS-*syn* can interconvert *via* a second-order saddle point, where the nitrogen is in an sp^2^ hybridized state.

**Fig. 2:**
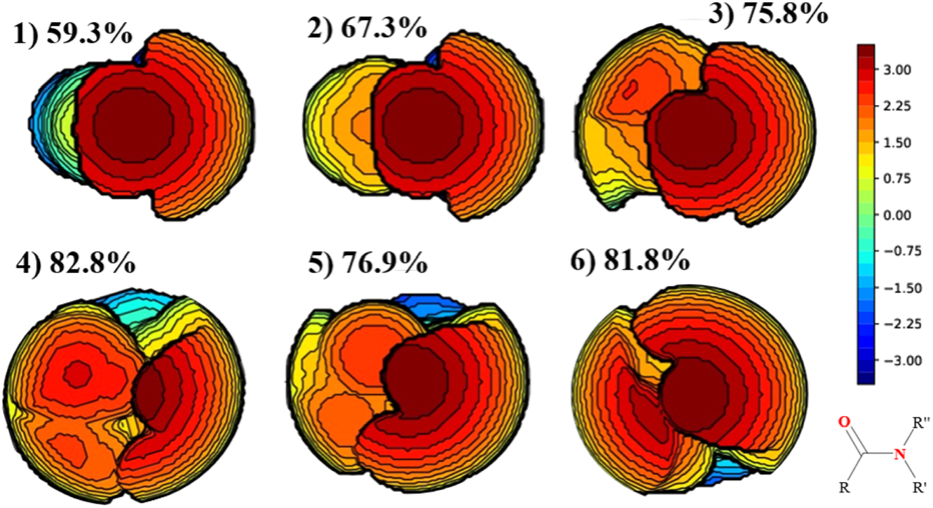
Topographic steric maps (in the *xy* plane) and % *V*_Bur_ values were generated for the *N*,*N*-Boc_2_ amides (entries 1–6), with a sphere radius of 3.5 Å. % *V*_Bur_ Represents the percent of buried volume. The centre of the sphere is defined by the midpoint between the two substituents, *R* and *R*′, with the *Z* axis passing through this point. The Boc moiety's carbon atom, bonded to the nitrogen, establishes the *xyz* plane. The steric maps are presented with isocontour curves measured in Å for a radius of 3.5 Å, providing a detailed visualization of the steric environment surrounding the amide structures.

However, in this class of amides, the C=N bond rotation behaves differently. The presence of *N*,*N*-Boc_2_ groups and the resulting delocalization of the nitrogen lone pair onto the Boc carbonyl make the rotation transition state more akin to the expected second-order saddle point, particularly for entries 1–5. The highest energy barrier (Δ*G*^‡^ = 8.5 kcal mol^−1^) is observed for *N*,*N*-Boc_2_ formamide (entry 1), where the substituent at the *C* position is a simple hydrogen atom. This causes the amide to remain nearly planar, as indicated by the Winkler–Dunitz parameters (*τ* = 1.9° and *χ*_N_ = 1.4). This planarity strengthens the C=N bond, making rotation more difficult, since amide bonds tend to be planar for the resonance stabilization due to the nitrogen lone pair and the carbonyl group.^48^ Nevertheless, this planarity can be disrupted with more bulky substituents. This effect was comprehensively studied by means of % *V*_Bur_ index of Cavallo and coworkers.^49,50^ In detail, as progressively bulkier substituents are introduced (entries 2–5), steric clashes between the substituents increase (Fig. 2), leading to a corresponding increase in the twist angle *τ* (reaching 73.5° with a *tert*-butyl group in entry 4) and making rotation around the C–N bond easier. The combination of intermediate and transition-state stabilization significantly lowers the rotation barrier, particularly in the case of the *tert*-butyl group (entry 4).

**Fig. 3:**
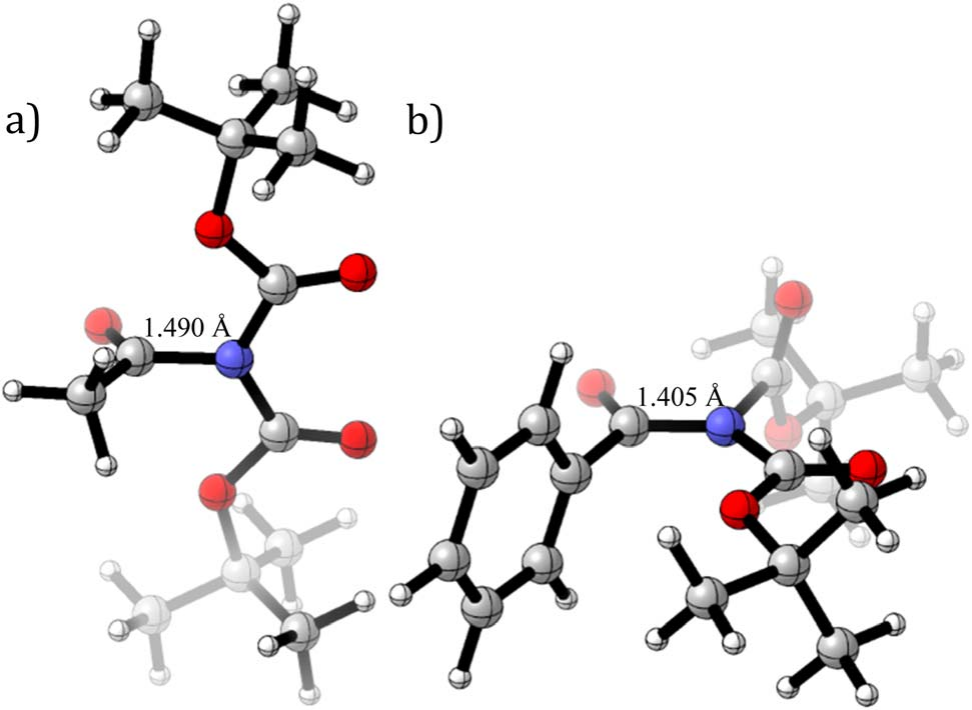
C–N rotation transition states for (a) entries 2 and (b) 6 in Table 1.

On the other hand, *N*,*N*-Boc_2_ benzamides (entries 6–13) behave differently.^51^ Rotation around the C–N bond induces rotation of the phenyl group around the C(phenyl)–C(carbonyl) bond. This allows the amide bond to remain planar while minimizing steric clashes between the phenyl ring and the *tert*-butoxy group, as illustrated in Fig. 3.

**Fig. 4:**
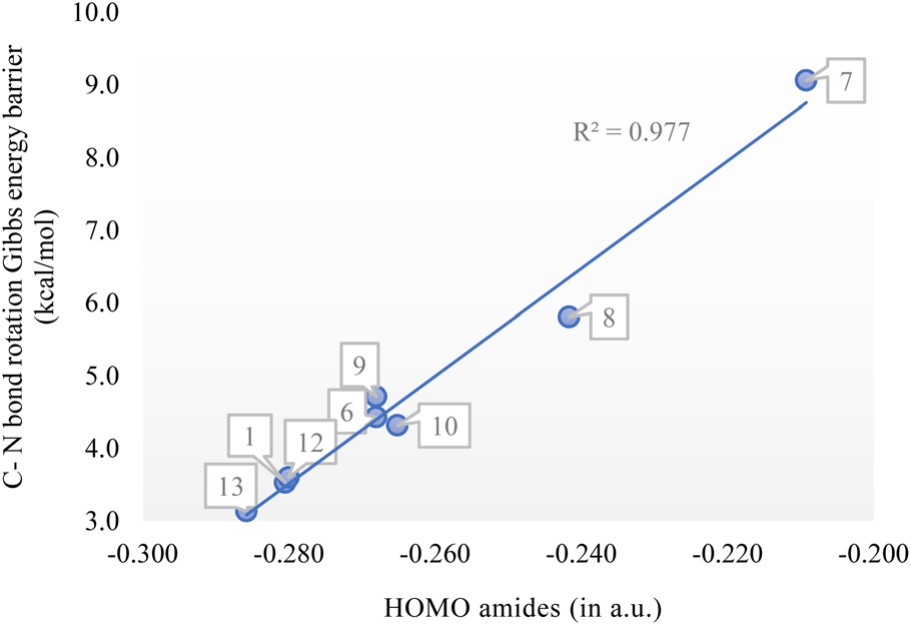
Correlation between the C–N bond rotation barrier (relative Gibbs energies in kcal mol^−1^) with the HOMO of the amides (in a.u.).

As a result, in transition state (TS) structures the twist angle (*τ*) for entries 6–13 is close to 0.0°, and the destabilization caused by increased steric hindrance is balanced by the partial restoration of amide planarity. This leads to relatively moderate activation energy barriers for rotation. Similar to *τ*, the rotation is hindered by electron-donating groups (EDGs) in the para position of the phenyl ring, with barriers increasing by 4.7 and 1.4 kcal mol^−1^ for entries 7 and 8, respectively, compared to the unsubstituted phenyl (entry 4). In contrast, halides have minimal impact, with rotation barrier variations of less than 1 kcal mol^−1^, while electron-withdrawing groups (EWGs) facilitate rotation, decreasing the barrier by up to 3.1 kcal mol^−1^ in the presence of a nitro group (entry 13). In terms of steric hindrance, % *V*_Bur_ remains constant, indicating that steric effects do not contribute to the decrease in the rotation barrier. However, a strong correlation with the amides' HOMO energies was observed (*R*^2^ = 0.977), as shown in Fig. 4.^52^

## Conclusions

In conclusion, this study provides a computational blueprint for understanding C=N bond rotation in *N*,*N*-Boc_2_ amides and its dependence on steric and electronic effects. In detail, the presence of bulky di-*tert*-butoxy groups induces significant twisting of the C=N bond, which is crucial for promoting N–C bond cleavage, enhancing the reactivity of these amides in cross-coupling reactions. The rotational barriers were found to vary significantly, with the highest being observed in *N*,*N*-Boc_2_ formamide due to its near-planar geometry. As bulkier substituents are introduced, steric clashes increase, resulting in higher twist angles and lower rotational barriers. In contrast, benzamides exhibited lower *τ* values and moderate activation barriers, with electron-donating groups increasing the barriers and electron-withdrawing groups lowering them. Notably, the % *V*_Bur_ values remained constant, indicating that steric hindrance does not contribute to the rotational barriers.

Importantly, this study identifies a strong correlation between HOMO energy levels and rotational barriers, suggesting that electronic effects are a key determinant of bond rotation. These findings provide valuable insights for the rational design of twisted amides in synthetic and pharmaceutical chemistry.

Furthermore, this work builds on past experimental data^34,35,53^ to provide a computational framework for amide bond distortion, addressing a long-standing challenge in amide activation.

## Data availability

The data supporting the findings of this study are available within the article and its ESI.†

## Conflicts of interest

There are no conflicts to declare.

## Supplementary Material

RA-015-D5RA00229J-s001

RA-015-D5RA00229J-s002
