# Supplementary material for: Towards the activity of twisted acyclic amides
Source: RSC Adv. 2025 Mar 24;15(11):8207–12. doi: 10.1039/d5ra00229j (PMC11932378; doi:10.1039/d5ra00229j)
Supplement: RA-015-D5RA00229J-s002 [file RA-015-D5RA00229J-s002.pdf]

Supplementary Information (SI) for RSC Advances.
This journal is © The Royal Society of Chemistry 2025

Towards the Activity of Twisted Acyclic Amides

Michele Tomasini,[a,b] Lucia Caporaso,[ab] Michal Szostak*,[c] and Albert Poater*,[a]

[a] *Institut de Química Computacional i Catàlisi, Departament de Química, Universitat de Girona, c/ Mª Aurèlia Capmany 69, 17003 Girona, Catalonia, Spain; orcid.org/0000-0002-8997-2599*

[b] *Dipartimento di Chimica e Biologia, Università di Salerno, Via Ponte don Melillo, 84084, Fisciano, Italy*

[c] Department of Chemistry, Rutgers University, 73 Warren Street, Newark, New Jersey 07102, United States

E-mail: michal.szostak@rutgers.edu, albert.poater@udg.edu

Table S1. Xyz coordinates and absolute energies (in a.u. of all computed species). The IRC and linear transit calculations are omitted for the sake of clarity).

37

Entry 1 SCF Done: -861.8429567 A.U.

O	0.787143000	2.879756000	0.311569000
O	1.578766000	0.388703000	-1.774437000
O	1.912706000	-0.074078000	0.427174000
O	-0.915242000	-1.265380000	-0.555746000
O	-2.270771000	0.438178000	0.120300000
N	-0.069597000	0.821667000	-0.186970000
C	-0.164918000	2.139953000	0.258354000
C	1.245332000	0.349092000	-0.627227000
C	-1.111558000	-0.119582000	-0.233058000
C	3.311688000	-0.580414000	0.300555000
C	3.656927000	-0.925702000	1.745791000
H	2.971766000	-1.682731000	2.132922000
H	3.589896000	-0.037714000	2.377553000
H	4.675628000	-1.316546000	1.797810000
C	3.316676000	-1.823435000	-0.586927000
H	2.599021000	-2.557860000	-0.214685000
H	4.313574000	-2.271116000	-0.565784000
H	3.066581000	-1.577062000	-1.617734000
C	4.203995000	0.538552000	-0.233158000
H	5.246765000	0.214762000	-0.183725000

H	4.089542000	1.437166000	0.376748000
H	3.963131000	0.781738000	-1.266886000
C	-3.530377000	-0.356159000	0.157382000
C	-4.550434000	0.685316000	0.609346000
H	-4.283484000	1.085003000	1.589996000
H	-5.539623000	0.227236000	0.677836000
H	-4.597916000	1.511184000	-0.103499000
C	-3.396339000	-1.480352000	1.183434000
H	-2.667350000	-2.223519000	0.864814000
H	-4.366066000	-1.969606000	1.305205000
H	-3.094020000	-1.075550000	2.152337000
C	-3.849665000	-0.871294000	-1.245053000
H	-3.864610000	-0.044018000	-1.958556000
H	-4.839351000	-1.335094000	-1.238063000
H	-3.119450000	-1.609488000	-1.572321000
H	-1.185634000	2.418276000	0.547317000

39

Entry_02 SCF Done: -901.1771417 A.U.

O	-1.000373	2.713072	0.009983
O	-1.639398	0.186998	1.806164
O	-1.954639	-0.135722	-0.422744
O	0.733350	-1.425368	0.454816
O	2.220748	0.218085	-0.046464
N	0.026401	0.727239	0.262261
C	0.047508	2.105132	-0.051254
C	-1.297588	0.226339	0.661569
C	1.012976	-0.269871	0.234924
C	-3.346779	-0.659668	-0.335690
C	-3.682654	-0.918512	-1.801420
H	-2.988474	-1.644128	-2.230561
H	-3.620177	0.007448	-2.376764

H	-4.697584	-1.314156	-1.883457
C	-3.347068	-1.956094	0.472440
H	-2.617393	-2.657580	0.062071
H	-4.338963	-2.411821	0.414583
H	-3.107401	-1.771552	1.518469
C	-4.253273	0.415863	0.260451
H	-5.292814	0.086155	0.185963
H	-4.143092	1.351376	-0.291900
H	-4.018329	0.597873	1.307981
C	3.409889	-0.671995	-0.129213
C	4.523383	0.315777	-0.469893
H	4.316019	0.819135	-1.416662
H	5.473489	-0.215308	-0.559894
H	4.620578	1.071057	0.312846
C	3.214452	-1.689381	-1.252584
H	2.420798	-2.395196	-1.014317
H	4.145487	-2.242915	-1.398966
H	2.969857	-1.179388	-2.187658
C	3.654422	-1.330413	1.227851
H	3.716540	-0.571388	2.011390
H	4.604985	-1.869160	1.197231
H	2.860688	-2.032516	1.476437
C	1.342296	2.770165	-0.443624
H	2.085155	2.675786	0.348652
H	1.765577	2.306797	-1.335288
H	1.117357	3.818249	-0.631367

45

Entry_03 SCF Done: -979.832975 A.U.

O	-0.991393	2.305607	0.675577
O	-1.768451	-0.557113	1.827580
O	-2.127193	-0.224848	-0.393214

O	0.482287	-1.916099	0.134782
O	2.106177	-0.331082	0.202287
N	-0.077816	0.266621	0.442071
C	0.012702	1.680673	0.405470
C	-1.433369	-0.226323	0.728868
C	0.848481	-0.768622	0.240494
C	-3.566979	-0.605829	-0.403298
C	-3.937270	-0.415456	-1.871313
H	-3.326757	-1.060682	-2.506585
H	-3.779798	0.622077	-2.173124
H	-4.988996	-0.668824	-2.023043
C	-3.704877	-2.067127	0.020369
H	-3.062581	-2.701449	-0.594551
H	-4.742050	-2.382862	-0.119575
H	-3.435500	-2.203240	1.066591
C	-4.347909	0.352379	0.494631
H	-5.417743	0.169559	0.364591
H	-4.133347	1.386932	0.218973
H	-4.093909	0.210221	1.543879
C	3.238288	-1.254469	-0.073151
C	4.432005	-0.302007	-0.061970
H	4.320172	0.463343	-0.833091
H	5.351645	-0.858705	-0.254808
H	4.521069	0.190379	0.908764
C	3.058806	-1.894008	-1.449517
H	2.204299	-2.568238	-1.465260
H	3.958656	-2.461675	-1.699464
H	2.920401	-1.122938	-2.211542
C	3.343692	-2.286265	1.048483
H	3.398688	-1.786097	2.018265
H	4.257044	-2.871147	0.911924

H	2.489594	-2.961022	1.046021
C	1.303314	2.360428	-0.030784
H	2.136769	1.870963	0.470614
C	1.271619	3.841762	0.355945
H	1.145272	3.968420	1.433055
H	2.210553	4.316768	0.059098
H	0.446651	4.359373	-0.136521
C	1.477564	2.180427	-1.552808
H	1.513570	1.128498	-1.838030
H	0.653425	2.657565	-2.090262
H	2.409784	2.651341	-1.875024

48

Entry_04 SCF Done: -1019.157785 A.U.

O	0.296240	1.065074	-1.782650
O	1.405622	-2.261980	0.144702
O	2.255934	-0.149575	0.110735
O	-1.299562	-2.054170	0.867978
O	-2.158303	-0.260430	-0.246422
N	0.048597	-0.381148	0.000665
C	0.043836	0.964152	-0.616926
C	1.276065	-1.070914	0.089998
C	-1.171110	-1.022364	0.268534
C	3.675538	-0.540167	-0.066149
C	4.381937	0.812925	-0.112455
H	4.229178	1.359513	0.820684
H	3.998511	1.416835	-0.937199
H	5.454762	0.664701	-0.254581
C	4.135699	-1.362565	1.137048
H	3.941838	-0.817148	2.063997
H	5.212050	-1.538869	1.061845
H	3.622682	-2.321851	1.177329

C	3.839445	-1.287231	-1.389915
H	4.903882	-1.437780	-1.586900
H	3.416176	-0.699941	-2.208163
H	3.350414	-2.259776	-1.360913
C	-3.576743	-0.689373	-0.157127
C	-4.311153	0.442131	-0.872467
H	-4.191103	1.384762	-0.334881
H	-5.377493	0.211589	-0.926426
H	-3.927566	0.566825	-1.886963
C	-4.006246	-0.781209	1.307384
H	-3.484121	-1.583938	1.824798
H	-5.081302	-0.973890	1.352263
H	-3.810127	0.162724	1.822043
C	-3.752906	-2.011229	-0.905101
H	-3.378312	-1.918052	-1.927187
H	-4.816582	-2.258875	-0.949895
H	-3.225160	-2.822222	-0.406281
C	-0.207661	2.170642	0.297733
C	1.133557	2.944027	0.352501
H	1.463377	3.213028	-0.652554
H	0.995839	3.859362	0.934481
H	1.912459	2.342967	0.822598
C	-0.631529	1.787139	1.722052
H	-1.590840	1.268400	1.731306
H	0.111579	1.148914	2.204323
H	-0.736861	2.694638	2.322370
C	-1.279391	3.055174	-0.368171
H	-2.233042	2.529046	-0.424729
H	-1.417833	3.967930	0.217480
H	-0.978068	3.330975	-1.379887

39

Entry_05 SCF Done: -1198.978224 A.U.

O	0.873812	1.713424	-1.267926
O	1.561672	-1.872591	-0.731538
O	2.435538	-0.052836	0.316722
O	-0.824504	-1.867931	1.042190
O	-1.977593	-0.498507	-0.367053
N	0.212272	-0.112451	-0.040257
C	0.118913	1.208353	-0.483393
C	1.482520	-0.798943	-0.207207
C	-0.913952	-0.936055	0.294569
C	3.877658	-0.376740	0.100872
C	4.570437	0.810386	0.762211
H	4.303676	0.869714	1.819394
H	4.278442	1.742844	0.275333
H	5.653668	0.695526	0.681390
C	4.207165	-1.687719	0.810725
H	3.924695	-1.630573	1.864421
H	5.284421	-1.863357	0.752669
H	3.689752	-2.528016	0.350182
C	4.162833	-0.421720	-1.399030
H	5.242044	-0.499163	-1.551934
H	3.809070	0.493945	-1.877547
H	3.684380	-1.278299	-1.872129
C	-3.337173	-1.070902	-0.120629
C	-4.214965	-0.204919	-1.018669
H	-4.155374	0.842771	-0.720389
H	-5.253287	-0.535181	-0.942677
H	-3.897222	-0.287799	-2.059952
C	-3.706017	-0.899720	1.351578
H	-3.076689	-1.512949	1.995046
H	-4.746251	-1.204033	1.490939

H	-3.610126	0.145930	1.649326
C	-3.343647	-2.530608	-0.568485
H	-3.007767	-2.610953	-1.604761
H	-4.364288	-2.916687	-0.506909
H	-2.699119	-3.141556	0.061651
C	-0.968176	2.119444	0.180202
F	-0.401933	3.297128	0.478554
F	-1.990121	2.354355	-0.658060
F	-1.464712	1.611559	1.326408

46

Entry_06 SCF Done: -1092.971312 A.U.

N	-0.490993	-0.409448	-0.075300
C	-0.170268	0.802975	-0.781845
O	-0.810326	1.142418	-1.747106
C	0.946043	1.612800	-0.218667
C	1.242981	1.606016	1.147879
C	1.675598	2.431661	-1.086486
C	2.269658	2.407092	1.640233
H	0.666502	0.984881	1.823077
C	2.713534	3.215190	-0.594960
H	1.422354	2.434297	-2.139609
C	3.011602	3.204405	0.769291
H	2.490807	2.408191	2.701365
H	3.288244	3.837518	-1.271275
H	3.817303	3.820434	1.152590
C	-1.857889	-0.826776	-0.005006
O	-2.215443	-1.960455	-0.168867
O	-2.606443	0.234493	0.281882
C	0.511232	-1.341403	0.301672
O	0.374913	-2.142928	1.186522
O	1.583460	-1.159974	-0.473378

C	2.875452	-1.833090	-0.187477
C	2.714815	-3.336778	-0.401845
H	3.690958	-3.820215	-0.309419
H	2.036654	-3.767285	0.333508
H	2.324918	-3.536120	-1.402664
C	3.342746	-1.485194	1.225843
H	2.687119	-1.918340	1.979947
H	4.351119	-1.880089	1.373041
H	3.375042	-0.401549	1.357104
C	3.794854	-1.211122	-1.235734
H	4.799027	-1.631282	-1.143874
H	3.420808	-1.415041	-2.241132
H	3.851370	-0.129131	-1.098379
C	-4.083719	0.194975	0.133593
C	-4.441038	-0.217764	-1.294135
H	-4.194348	-1.262056	-1.481087
H	-5.514567	-0.080938	-1.446655
H	-3.904071	0.407867	-2.010399
C	-4.469490	1.647974	0.398235
H	-4.151291	1.952095	1.397797
H	-3.996512	2.306103	-0.333329
H	-5.553605	1.762335	0.327024
C	-4.674978	-0.740895	1.186545
H	-5.765942	-0.680239	1.149434
H	-4.373015	-1.771982	1.009336
H	-4.347995	-0.442157	2.185439

54

Entry_07 SCF Done: -1226.991137 A.U.

N	1.361815	0.552394	0.059951
C	0.639191	-0.281086	1.018670
O	1.166359	-0.590688	2.060450

C	-0.707676	-0.703910	0.605526
C	-1.132137	-0.698336	-0.729029
C	-1.606503	-1.153266	1.582673
C	-2.406149	-1.109583	-1.080247
H	-0.450637	-0.382200	-1.509779
C	-2.887255	-1.553209	1.252125
H	-1.281313	-1.173241	2.616037
C	-3.331198	-1.535472	-0.095304
H	-2.680538	-1.103411	-2.125423
H	-3.547773	-1.881348	2.042080
C	0.815784	1.774008	-0.390600
O	1.153689	2.339123	-1.397073
O	-0.132118	2.161000	0.473199
C	2.718074	0.246997	-0.226011
O	3.571456	1.067417	-0.428404
O	2.850449	-1.082736	-0.240513
C	4.186270	-1.720515	-0.194692
C	4.952359	-1.216783	1.028966
H	5.860830	-1.811851	1.152624
H	5.234348	-0.170485	0.919135
H	4.338373	-1.326628	1.925633
C	4.925812	-1.453899	-1.505313
H	5.151511	-0.394739	-1.618729
H	5.861713	-2.019204	-1.513154
H	4.320018	-1.780102	-2.354331
C	3.830536	-3.198143	-0.047258
H	4.742892	-3.798041	-0.009664
H	3.261903	-3.361864	0.870319
H	3.227279	-3.531838	-0.894461
C	-1.071327	3.259764	0.156294
C	-0.305700	4.580762	0.099978

H	0.390500	4.593388	-0.737314
H	-1.014482	5.405396	-0.014387
H	0.252823	4.732135	1.026636
C	-2.024966	3.215489	1.348458
H	-2.520250	2.243460	1.401771
H	-1.478584	3.377557	2.279871
H	-2.784045	3.994700	1.246832
C	-1.812589	2.953013	-1.145855
H	-2.594648	3.701900	-1.295254
H	-1.139168	2.978385	-2.001559
H	-2.282439	1.968546	-1.088488
N	-4.609124	-1.915783	-0.431284
C	-5.500998	-2.458712	0.583047
H	-6.461135	-2.693151	0.127035
H	-5.102308	-3.377281	1.032025
H	-5.680654	-1.734615	1.384609
C	-5.011851	-1.967725	-1.828911
H	-6.059830	-2.255713	-1.889351
H	-4.906979	-0.989093	-2.308598
H	-4.423001	-2.696230	-2.401153

50

Entry_08 SCF Done: -1207.535222 A.U.

N	1.082821	0.519865	0.070574
C	0.416695	-0.466537	0.897006
O	0.943417	-0.870169	1.905962
C	-0.892316	-0.948690	0.401698
C	-1.253270	-0.891853	-0.953021
C	-1.786396	-1.508194	1.316742
C	-2.478958	-1.373850	-1.375585
H	-0.563466	-0.479991	-1.679721
C	-3.028510	-1.980623	0.908559

H	-1.499760	-1.559667	2.360112
C	-3.380323	-1.913676	-0.446473
H	-2.766178	-1.346658	-2.419404
H	-3.706100	-2.395363	1.641967
C	0.425256	1.705305	-0.339478
O	0.768370	2.371762	-1.279170
O	-0.613216	1.924987	0.474016
C	2.496581	0.431154	-0.096618
O	3.233674	1.378427	-0.091118
O	2.816825	-0.850886	-0.269407
C	4.223591	-1.310743	-0.167192
C	4.799224	-0.894731	1.186582
H	5.768914	-1.379169	1.326421
H	4.938022	0.183926	1.246164
H	4.131323	-1.211650	1.990568
C	5.026109	-0.761198	-1.345633
H	5.107085	0.323404	-1.293071
H	6.030560	-1.192698	-1.329686
H	4.548984	-1.035898	-2.289525
C	4.071017	-2.827196	-0.255587
H	5.053167	-3.302532	-0.201076
H	3.454451	-3.195646	0.566707
H	3.598311	-3.109317	-1.198922
C	-1.632902	2.958461	0.172987
C	-0.993421	4.341772	0.277768
H	-0.247357	4.487921	-0.501957
H	-1.768097	5.106361	0.175200
H	-0.515439	4.463798	1.252516
C	-2.654706	2.727275	1.283969
H	-3.056799	1.713470	1.224711
H	-2.190529	2.860097	2.263374

H	-3.477018	3.439779	1.186163
C	-2.251455	2.697867	-1.200746
H	-3.087331	3.386001	-1.350106
H	-1.527290	2.853456	-1.999170
H	-2.632895	1.676003	-1.255993
O	-4.562024	-2.344379	-0.956704
C	-5.525223	-2.917938	-0.076576
H	-6.373686	-3.187325	-0.702825
H	-5.132865	-3.816593	0.411088
H	-5.848079	-2.198347	0.683401

46

Entry_09 SCF Done: -1192.240212 A.U.

N	0.747170	-0.488377	0.073664
C	0.273801	0.611736	0.871857
O	0.881212	0.971475	1.850948
C	-0.959394	1.287944	0.386668
C	-1.309057	1.307060	-0.967617
C	-1.758547	1.958131	1.319529
C	-2.451228	1.978775	-1.390467
H	-0.683367	0.806003	-1.696053
C	-2.912140	2.616824	0.914254
H	-1.467441	1.946831	2.362449
C	-3.234328	2.612126	-0.436734
H	-2.738404	2.017193	-2.433431
H	-3.555868	3.130379	1.616924
C	2.159052	-0.687382	-0.055924
O	2.687699	-1.761716	0.018804
O	2.728762	0.492701	-0.282371
C	-0.115572	-1.537158	-0.340084
O	0.111614	-2.253141	-1.277895
O	-1.179213	-1.566408	0.467028

C	-2.359911	-2.417449	0.170436
C	-1.960211	-3.886985	0.286046
H	-2.852135	-4.511419	0.188286
H	-1.249553	-4.162682	-0.491667
H	-1.509708	-4.080212	1.262283
C	-2.922484	-2.065932	-1.206766
H	-2.231888	-2.343370	-2.001870
H	-3.860007	-2.607293	-1.355682
H	-3.131387	-0.995720	-1.268684
C	-3.329194	-2.010704	1.277564
H	-4.258534	-2.576844	1.182945
H	-2.894927	-2.212329	2.258881
H	-3.558484	-0.944927	1.210966
C	4.200455	0.668291	-0.175407
C	4.666877	0.219616	1.209276
H	4.585374	-0.860066	1.327482
H	5.712971	0.506303	1.343138
H	4.069721	0.707881	1.982457
C	4.354454	2.177349	-0.345041
H	3.957883	2.496136	-1.311449
H	3.816203	2.705803	0.444301
H	5.411178	2.449636	-0.294503
C	4.885898	-0.094341	-1.307852
H	5.956080	0.128625	-1.294887
H	4.748564	-1.168879	-1.197357
H	4.481719	0.217611	-2.273903
F	-4.354399	3.253252	-0.838534

46

Entry_10 SCF Done: -1552.596927 A.U.

N	1.058500	-0.477657	0.070822
C	0.455199	0.516401	0.914400

O	1.036909	0.947782	1.880191
C	-0.888294	1.002058	0.493908
C	-1.279754	1.023441	-0.847657
C	-1.757685	1.486301	1.475721
C	-2.531830	1.509338	-1.208308
H	-0.604276	0.667282	-1.616108
C	-3.018543	1.955157	1.130579
H	-1.437826	1.479274	2.510386
C	-3.394505	1.959470	-0.212185
H	-2.838751	1.536848	-2.245745
H	-3.704657	2.315187	1.886197
C	2.482485	-0.473671	-0.089899
O	3.156008	-1.465519	-0.049368
O	2.876815	0.778096	-0.299732
C	0.343544	-1.625001	-0.366812
O	0.647753	-2.264677	-1.337212
O	-0.682077	-1.838446	0.460610
C	-1.736292	-2.840396	0.155617
C	-1.125502	-4.239244	0.204497
H	-1.920162	-4.982802	0.101506
H	-0.403979	-4.380580	-0.598861
H	-0.625381	-4.399912	1.162300
C	-2.380553	-2.524743	-1.194097
H	-1.677976	-2.666635	-2.014065
H	-3.231276	-3.193717	-1.345574
H	-2.747395	-1.496247	-1.207625
C	-2.723801	-2.617700	1.298311
H	-3.564402	-3.308410	1.201260
H	-2.239115	-2.789522	2.261529
H	-3.105500	-1.594591	1.278125
C	4.311892	1.155934	-0.215432

C	4.865505	0.748033	1.149562
H	4.938631	-0.334639	1.243361
H	5.863695	1.175997	1.271360
H	4.222249	1.131063	1.944722
C	4.249920	2.674857	-0.352053
H	3.791977	2.955120	-1.303142
H	3.660370	3.106090	0.459419
H	5.259138	3.091308	-0.314580
C	5.072515	0.520360	-1.377836
H	6.101227	0.890369	-1.378921
H	5.088757	-0.564904	-1.290239
H	4.608482	0.792821	-2.328800
Cl	-4.987789	2.545455	-0.656357

49

Entry_11 SCF Done: -1430.12323 A.U.

N	1.565805	0.438033	0.089491
C	0.842688	-0.446035	0.948735
O	1.378481	-0.987794	1.885127
C	-0.583793	-0.701773	0.584047
C	-1.008995	-0.709876	-0.746700
C	-1.489393	-0.987395	1.608686
C	-2.336888	-0.986012	-1.051745
H	-0.303643	-0.503625	-1.542505
C	-2.821099	-1.242358	1.309444
H	-1.139794	-0.992478	2.633408
C	-3.242760	-1.235769	-0.021619
H	-2.671429	-0.994064	-2.081286
H	-3.533320	-1.438648	2.100837
C	1.021537	1.669092	-0.370784
O	1.419490	2.238423	-1.350732
O	0.037622	2.044221	0.447953

C	2.980096	0.235311	-0.072882
O	3.783320	1.122739	-0.002661
O	3.193090	-1.051124	-0.318680
C	4.563041	-1.629233	-0.256915
C	5.171219	-1.345167	1.115969
H	6.100211	-1.911705	1.217514
H	5.395261	-0.286802	1.242507
H	4.483047	-1.659554	1.903534
C	5.400357	-1.068982	-1.404981
H	5.569089	-0.000061	-1.283825
H	6.367095	-1.578914	-1.425662
H	4.899602	-1.243426	-2.360286
C	4.288174	-3.119135	-0.440097
H	5.229321	-3.673402	-0.422979
H	3.646814	-3.489622	0.362020
H	3.792982	-3.302422	-1.396105
C	-0.853169	3.191423	0.125324
C	-0.036552	4.481108	0.163710
H	0.702488	4.501731	-0.635930
H	-0.708170	5.335534	0.046538
H	0.476589	4.575411	1.123421
C	-1.867875	3.133317	1.264177
H	-2.402890	2.181260	1.251053
H	-1.367023	3.238186	2.228730
H	-2.592000	3.943829	1.156131
C	-1.530942	2.962057	-1.225185
H	-2.270213	3.750079	-1.388113
H	-0.811601	2.987653	-2.042395
H	-2.049765	2.001201	-1.231361
C	-4.675517	-1.560591	-0.350371
F	-5.081090	-0.971652	-1.498160

F	-4.861031	-2.894987	-0.509915
F	-5.523444	-1.164249	0.625560

47

Entry_12 SCF Done: -1185.23895 A.U.

N	0.957145	-0.437451	0.084396
C	0.385586	0.593614	0.890570
O	0.984616	1.065211	1.826606
C	-0.959758	1.092044	0.470722
C	-1.342196	1.124504	-0.873312
C	-1.820061	1.586696	1.454914
C	-2.582869	1.632693	-1.233787
H	-0.667490	0.758221	-1.637505
C	-3.069887	2.076650	1.107061
H	-1.499287	1.571445	2.488956
C	-3.457472	2.099281	-0.242119
H	-2.881487	1.667335	-2.274059
H	-3.748742	2.444182	1.866424
C	2.388942	-0.500621	-0.040268
O	3.013911	-1.516349	0.081881
O	2.838615	0.716016	-0.318486
C	0.209193	-1.566736	-0.351314
O	0.519676	-2.234690	-1.299862
O	-0.845325	-1.726181	0.449857
C	-1.918153	-2.711161	0.143344
C	-1.343262	-4.121941	0.246885
H	-2.153423	-4.848332	0.143116
H	-0.605212	-4.303816	-0.532932
H	-0.872633	-4.267972	1.221761
C	-2.517058	-2.417022	-1.231545
H	-1.796744	-2.598731	-2.027853
H	-3.380205	-3.068400	-1.388455

H	-2.858159	-1.380855	-1.283219
C	-2.928612	-2.432509	1.252885
H	-3.783617	-3.104537	1.151958
H	-2.473956	-2.589875	2.233025
H	-3.284714	-1.401586	1.195441
C	4.290031	1.037183	-0.236555
C	4.805969	0.695956	1.160758
H	4.828823	-0.380292	1.326566
H	5.820957	1.085989	1.269922
H	4.171069	1.159265	1.919001
C	4.295860	2.544688	-0.473403
H	3.864045	2.780951	-1.448318
H	3.715331	3.053853	0.298446
H	5.321745	2.918910	-0.446629
C	5.034982	0.293268	-1.343117
H	6.078775	0.617879	-1.353219
H	5.003240	-0.783520	-1.184100
H	4.595000	0.521612	-2.316793
C	-4.747048	2.601022	-0.606694
N	-5.790057	3.001816	-0.900348

48

Entry_13 SCF Done: -1297.538068 A.U.

N	-1.265710	0.433772	0.109554
C	-0.603737	-0.535621	0.916468
O	-1.167863	-1.086277	1.831111
C	0.799722	-0.880416	0.526976
C	1.204483	-0.888621	-0.811095
C	1.691835	-1.258979	1.534003
C	2.502075	-1.255327	-1.145600
H	0.505254	-0.613273	-1.591058
C	2.997014	-1.607852	1.215446

H	1.353432	-1.266221	2.562263
C	3.381133	-1.595892	-0.122892
H	2.838968	-1.275245	-2.172657
H	3.712577	-1.886495	1.976388
C	-2.699975	0.358533	-0.014480
O	-3.419716	1.304471	0.138852
O	-3.027309	-0.886722	-0.329059
C	-0.634714	1.630133	-0.333760
O	-1.021756	2.262515	-1.278533
O	0.407514	1.893999	0.454805
C	1.373377	2.981744	0.135674
C	0.661980	4.327427	0.254327
H	1.393296	5.131641	0.139670
H	-0.103598	4.436098	-0.512543
H	0.195678	4.423632	1.237443
C	1.977122	2.753181	-1.249486
H	1.230984	2.866810	-2.034445
H	2.769898	3.486678	-1.414957
H	2.417773	1.755956	-1.312693
C	2.423446	2.801574	1.228552
H	3.204904	3.556552	1.118523
H	1.970653	2.908688	2.216286
H	2.880759	1.812058	1.160515
C	-4.441829	-1.349817	-0.266870
C	-4.993367	-1.103710	1.136728
H	-5.121718	-0.040479	1.335197
H	-5.965932	-1.593805	1.227071
H	-4.319246	-1.526364	1.884763
C	-4.300098	-2.842493	-0.550052
H	-3.843983	-3.005256	-1.528921
H	-3.675885	-3.317014	0.209694

H	-5.284908	-3.314973	-0.541597
C	-5.250638	-0.647205	-1.355300
H	-6.257572	-1.071729	-1.383373
H	-5.325462	0.421918	-1.162600
H	-4.786115	-0.800415	-2.332275
N	4.775668	-1.962803	-0.471602
O	5.529679	-2.260229	0.445435
O	5.088646	-1.940942	-1.654886

36

TS_Entry_01 SCF Done: -861.8295484 A.U.

N	0.000004	0.093087	0.058821
C	0.000029	1.546918	-0.149552
O	-0.000068	2.318671	0.756332
C	1.239372	-0.572056	0.101728
O	1.410556	-1.733363	0.341344
O	2.179495	0.347900	-0.191194
C	-1.239370	-0.572049	0.101732
O	-1.410548	-1.733356	0.341354
O	-2.179493	0.347898	-0.191202
C	-3.626938	0.026070	-0.128325
C	-3.990829	-0.412540	1.289923
H	-5.076321	-0.515742	1.365036
H	-3.531512	-1.367632	1.540257
H	-3.666760	0.339810	2.012829
C	-3.961812	-1.034538	-1.176521
H	-3.493161	-1.987789	-0.937496
H	-5.045426	-1.173598	-1.212715
H	-3.625793	-0.709979	-2.164453
C	-4.270442	1.367165	-0.471890
H	-5.358314	1.269557	-0.457109
H	-3.976889	2.129575	0.252483

H	-3.964173	1.696180	-1.467318
C	3.626941	0.026076	-0.128320
C	3.990834	-0.412543	1.289924
H	3.531513	-1.367635	1.540254
H	5.076325	-0.515750	1.365035
H	3.666769	0.339804	2.012836
C	4.270442	1.367176	-0.471876
H	3.964177	1.696193	-1.467304
H	3.976883	2.129582	0.252499
H	5.358314	1.269572	-0.457089
C	3.961817	-1.034523	-1.176524
H	5.045432	-1.173574	-1.212726
H	3.493175	-1.987779	-0.937502
H	3.625789	-0.709961	-2.164452
H	0.000148	1.834625	-1.209684

39

TS_Entry_02 SCF Done: -901.1722587 A.U.

N	0.000031	-0.027338	0.002541
C	0.000337	1.448294	0.205939
O	0.003779	1.891650	1.314419
C	1.235267	-0.696808	-0.037549
O	1.398574	-1.884709	-0.047865
O	2.193855	0.251616	-0.080855
C	-1.235232	-0.696438	-0.039653
O	-1.398634	-1.884262	-0.057264
O	-2.193894	0.252268	-0.075515
C	-3.631629	-0.097605	-0.000202
C	-3.911103	-0.839496	1.306810
H	-4.990160	-0.971239	1.420650
H	-3.436825	-1.819641	1.315510
H	-3.544269	-0.258329	2.156130

C	-4.030014	-0.908596	-1.233218
H	-3.546819	-1.884168	-1.236593
H	-5.113833	-1.051150	-1.235025
H	-3.754781	-0.371816	-2.144647
C	-4.297310	1.276763	-0.004239
H	-5.382137	1.163490	0.055035
H	-3.958931	1.867891	0.849138
H	-4.054577	1.818443	-0.921311
C	3.631543	-0.097538	-0.001288
C	3.909536	-0.830238	1.311223
H	3.435814	-1.810579	1.326065
H	4.988498	-0.960602	1.427489
H	3.541178	-0.243411	2.155981
C	4.297056	1.276855	-0.014074
H	4.055179	1.812173	-0.935102
H	3.957740	1.873771	0.834893
H	5.381837	1.164145	0.047058
C	4.031444	-0.917076	-1.228131
H	5.115244	-1.059808	-1.227539
H	3.548068	-1.892563	-1.225354
H	3.757439	-0.386593	-2.143601
C	-0.002896	2.238603	-1.067828
H	-0.004566	3.304268	-0.845066
H	0.881499	1.973774	-1.651558
H	-0.887246	1.970501	-1.650025

45

TS_Entry_03 SCF Done: -979.8294905 A.U.

N	-0.030577	-0.341972	-0.196490
C	0.025363	1.052044	-0.697015
O	0.310844	1.241188	-1.842912
C	-1.268048	-0.935138	0.086200

O	-1.434709	-2.005624	0.603725
O	-2.225009	-0.069558	-0.305847
C	1.189518	-1.025441	-0.030092
O	1.328435	-2.214697	0.029904
O	2.154251	-0.089726	0.059735
C	3.589801	-0.449129	0.003535
C	3.880602	-1.200719	-1.295376
H	4.960657	-1.331424	-1.399998
H	3.409235	-2.182480	-1.299939
H	3.517691	-0.627321	-2.151565
C	3.963670	-1.255032	1.247083
H	3.462970	-2.221855	1.253009
H	5.044898	-1.415907	1.261193
H	3.687015	-0.706514	2.150918
C	4.262956	0.921681	0.007258
H	5.348198	0.803021	-0.028924
H	3.944191	1.505369	-0.858831
H	4.003743	1.472854	0.914118
C	-3.661017	-0.350283	-0.077033
C	-4.070901	-1.597693	-0.859333
H	-3.587518	-2.488674	-0.462141
H	-5.154640	-1.724148	-0.792345
H	-3.802312	-1.487015	-1.912531
C	-4.327821	0.900207	-0.646344
H	-4.001186	1.791127	-0.104818
H	-4.074293	1.022122	-1.701313
H	-5.413000	0.817120	-0.553674
C	-3.928724	-0.481870	1.422610
H	-5.006409	-0.552093	1.590621
H	-3.451788	-1.370180	1.833492
H	-3.558125	0.399705	1.952177

C	-0.277585	2.155582	0.310302
C	0.521101	3.416229	-0.051005
H	0.220424	4.245960	0.593067
H	1.591371	3.242254	0.090867
H	0.359497	3.701825	-1.090512
C	-0.063952	1.736617	1.770241
H	0.970169	1.425624	1.934500
H	-0.276116	2.581714	2.429579
H	-0.717088	0.913239	2.064014
H	-1.343822	2.364485	0.161435

48

TS_Entry_04 SCF Done: -1019.157446 A.U.

N	0.002683	-0.421680	-0.062256
C	-0.000963	0.947230	-0.647884
O	0.004558	1.062747	-1.837546
C	-1.225654	-1.069412	0.147508
O	-1.373548	-2.210943	0.484697
O	-2.203519	-0.168524	-0.087481
C	1.233310	-1.059942	0.163287
O	1.383849	-2.189468	0.538112
O	2.208487	-0.165449	-0.102344
C	3.633948	-0.579644	-0.112987
C	3.836619	-1.688075	-1.146859
H	4.905698	-1.891938	-1.247453
H	3.333090	-2.606471	-0.850216
H	3.454580	-1.369646	-2.119729
C	4.060105	-0.999278	1.293754
H	3.557587	-1.913918	1.603014
H	5.140310	-1.167069	1.306640
H	3.829735	-0.206528	2.010087
C	4.350339	0.698154	-0.544522

H	5.419710	0.501185	-0.648425
H	3.963364	1.049554	-1.502964
H	4.218810	1.488900	0.196569
C	-3.627779	-0.586472	-0.111006
C	-3.828714	-1.653521	-1.187702
H	-3.320165	-2.580609	-0.928854
H	-4.897076	-1.858482	-1.293508
H	-3.450719	-1.295003	-2.148122
C	-4.349316	0.705301	-0.488534
H	-4.216387	1.465476	0.283859
H	-3.967747	1.095942	-1.433883
H	-5.418633	0.509851	-0.595627
C	-4.050231	-1.062844	1.278834
H	-5.130322	-1.231745	1.287103
H	-3.546438	-1.988534	1.550536
H	-3.819007	-0.299186	2.025830
C	-0.011153	2.135224	0.324297
C	1.263066	2.964301	0.049595
H	1.237568	3.876342	0.652084
H	2.156794	2.398517	0.308458
H	1.321294	3.242834	-1.004125
C	-1.249498	2.993515	-0.014494
H	-1.240874	3.282393	-1.066889
H	-2.168173	2.444122	0.186415
H	-1.240847	3.899416	0.597599
C	-0.052519	1.700587	1.795295
H	0.820638	1.101162	2.061504
H	-0.058724	2.587095	2.435052
H	-0.950836	1.120441	2.015841

39

TS_Entry_05 SCF Done: -1198.972985 A.U.

N	-0.000059	-0.445459	0.112946
C	-0.000046	0.873961	0.710778
O	-0.000294	1.090563	1.877870
C	1.249467	-1.080472	-0.112143
O	1.397526	-2.163494	-0.597648
O	2.192796	-0.231422	0.314230
C	-1.249597	-1.080624	-0.111617
O	-1.397684	-2.164218	-0.595838
O	-2.192913	-0.230987	0.313619
C	-3.641498	-0.508623	0.121347
C	-4.027605	-1.753321	0.918112
H	-5.112452	-1.880633	0.878656
H	-3.554980	-2.645823	0.510852
H	-3.733563	-1.639358	1.964094
C	-3.936609	-0.645199	-1.371371
H	-3.471901	-1.537538	-1.788481
H	-5.017604	-0.714034	-1.516422
H	-3.573129	0.232714	-1.910402
C	-4.285409	0.747218	0.702187
H	-5.372546	0.671551	0.628457
H	-4.013478	0.866314	1.752955
H	-3.958319	1.633474	0.154686
C	3.641375	-0.508832	0.121536
C	4.027498	-1.754479	0.916807
H	3.554676	-2.646455	0.508626
H	5.112320	-1.881882	0.876971
H	3.733692	-1.641674	1.962982
C	4.285324	0.746314	0.703832
H	3.958331	1.633210	0.157310
H	4.013331	0.864242	1.754715
H	5.372461	0.670660	0.630089

C	3.936433	-0.643640	-1.371355
H	5.017431	-0.712151	-1.516531
H	3.471835	-1.535558	-1.789485
H	3.572806	0.234845	-1.909352
C	0.000240	2.030545	-0.338103
F	0.000267	3.218307	0.268145
F	-1.085238	1.957920	-1.133011
F	1.085927	1.957739	-1.132721

46

TS_Entry_06 SCF Done: -1092.964997 A.U.

O	0.731930	-2.392007	-0.467336
O	0.583902	2.118541	-0.271829
O	-1.408937	1.035338	-0.126687
O	2.362206	-0.010861	-1.848637
O	2.592217	-0.152825	0.409885
N	0.497134	-0.148674	-0.453724
C	-0.022472	-1.449294	-0.347383
C	-1.485921	-1.652331	-0.089311
C	-0.095084	1.118620	-0.273844
C	1.939581	-0.093253	-0.733575
C	-2.373107	-1.757330	-1.159922
H	-2.014378	-1.621871	-2.173870
C	-3.717319	-2.029691	-0.919512
H	-4.406625	-2.111961	-1.752286
C	-4.175703	-2.197598	0.386852
C	-3.283278	-2.101649	1.453915
H	-3.633949	-2.240106	2.470456
C	-1.936946	-1.832940	1.217563
H	-1.239554	-1.761028	2.044549
C	-2.244883	2.236367	0.137417
C	-3.640153	1.628432	0.252983

H	-3.672707	0.893489	1.058977
H	-3.916269	1.124665	-0.674774
H	-4.369434	2.415213	0.458936
C	-1.809389	2.879375	1.453075
H	-1.825414	2.140215	2.257685
H	-2.509256	3.678841	1.709132
H	-0.808695	3.301920	1.376200
C	-2.151555	3.194730	-1.048461
H	-2.869949	4.006676	-0.908784
H	-2.400709	2.673380	-1.975736
H	-1.153191	3.619887	-1.137364
C	4.082780	-0.152253	0.443118
C	4.368392	-0.248347	1.938809
H	3.936888	0.604984	2.466216
H	5.447448	-0.255653	2.108721
H	3.942977	-1.165728	2.350849
C	4.598025	1.162735	-0.138885
H	4.393233	1.231591	-1.206141
H	5.678550	1.221293	0.015616
H	4.128013	2.009643	0.365778
C	4.602178	-1.380291	-0.302310
H	4.127745	-2.285019	0.083477
H	5.681584	-1.459110	-0.149058
H	4.405219	-1.309634	-1.370913
H	-5.222881	-2.408157	0.572044

54

TS_Entry_07 SCF Done: -1226.978174 A.U.

O	-1.159173	-2.488870	0.039077
O	-0.188380	1.164656	-1.877702
O	-0.119466	1.640230	0.342394
O	-3.050807	1.191321	-0.516980

O	-3.348004	-0.961454	0.147990
N	-1.261594	-0.203086	-0.325224
C	-0.593059	-1.435223	-0.111704
C	0.904035	-1.295510	-0.065703
C	-0.463050	0.951394	-0.731128
C	-2.643225	0.082081	-0.250611
C	1.544061	-1.079651	1.154951
H	0.956292	-0.980400	2.060646
C	2.926704	-0.985353	1.232292
H	3.377001	-0.824833	2.201498
C	3.729460	-1.089795	0.073378
C	3.067906	-1.327963	-1.152210
H	3.629653	-1.433460	-2.069153
C	1.684564	-1.425172	-1.213979
H	1.208493	-1.589928	-2.173509
C	0.740245	2.851381	0.233285
C	0.896885	3.261137	1.694864
H	1.377693	2.462534	2.263916
H	-0.077895	3.469857	2.140445
H	1.513344	4.160395	1.763060
C	2.087403	2.465668	-0.377932
H	2.526491	1.628293	0.167963
H	2.764736	3.320917	-0.308790
H	1.984768	2.185846	-1.424863
C	0.002326	3.919335	-0.571164
H	0.572631	4.851283	-0.535473
H	-0.986500	4.098866	-0.143786
H	-0.114433	3.619677	-1.611576
C	-4.824859	-0.892134	0.276880
C	-5.166356	-2.308707	0.732447
H	-4.832298	-3.037243	-0.008361

H	-6.246723	-2.404961	0.864561
H	-4.672862	-2.534040	1.679799
C	-5.445791	-0.585172	-1.085621
H	-5.197786	0.421980	-1.417064
H	-6.532781	-0.673861	-1.011410
H	-5.093338	-1.302918	-1.830119
C	-5.202485	0.137893	1.341310
H	-4.679408	-0.077337	2.276295
H	-6.277733	0.079953	1.529822
H	-4.955812	1.148514	1.019829
N	5.107345	-0.946511	0.134881
C	5.764109	-0.947221	1.433114
H	5.602560	-1.883047	1.987637
H	6.835619	-0.812996	1.291164
H	5.410316	-0.117682	2.051954
C	5.909994	-1.292326	-1.028662
H	5.638610	-0.674211	-1.888856
H	6.959262	-1.100691	-0.808075
H	5.803286	-2.348340	-1.316231

50

TS_Entry_08 SCF Done: -1207.526889 A.U.

O	0.919201	-2.358588	-0.481935
O	1.443223	2.120028	-0.265974
O	-0.692724	1.354863	-0.140454
O	2.887404	-0.241537	-1.851778
O	3.079539	-0.425429	0.407116
N	1.013691	-0.105705	-0.468242
C	0.306159	-1.317938	-0.364900
C	-1.169198	-1.293544	-0.113234
C	0.619561	1.234978	-0.280066
C	2.449325	-0.264573	-0.739613

C	-2.063208	-1.244826	-1.175693
H	-1.692360	-1.170593	-2.191648
C	-3.439387	-1.278721	-0.950490
H	-4.113210	-1.239127	-1.795254
C	-3.922678	-1.359372	0.358471
C	-3.022002	-1.424278	1.428900
H	-3.417287	-1.498444	2.434634
C	-1.656649	-1.395139	1.192238
H	-0.965075	-1.444937	2.025639
C	-1.333093	2.666794	0.137477
C	-2.807456	2.283052	0.237128
H	-2.961010	1.550883	1.031610
H	-3.151503	1.842268	-0.700138
H	-3.405958	3.171431	0.451442
C	-0.813001	3.215170	1.465289
H	-0.950962	2.476099	2.258202
H	-1.380791	4.110795	1.729924
H	0.242160	3.476504	1.400106
C	-1.084130	3.616957	-1.032665
H	-1.669708	4.527982	-0.884089
H	-1.403503	3.153835	-1.969356
H	-0.031465	3.883826	-1.109827
C	4.552579	-0.647097	0.448320
C	4.811416	-0.792888	1.944942
H	4.508381	0.112309	2.475351
H	5.876229	-0.961899	2.120832
H	4.251258	-1.638795	2.348694
C	5.261778	0.579649	-0.122422
H	5.075957	0.684046	-1.190277
H	6.338040	0.475932	0.038179
H	4.919678	1.484273	0.384999

C	4.888855	-1.934743	-0.301807
H	4.282807	-2.760977	0.075662
H	5.943623	-2.173744	-0.142990
H	4.711323	-1.829861	-1.371000
C	-6.213503	-1.326088	-0.342819
H	-6.123983	-2.183034	-1.019740
H	-7.182467	-1.358180	0.152629
H	-6.131136	-0.396630	-0.917799
O	-5.243778	-1.381635	0.696470

46

TS_Entry_09 SCF Done: -1192.233814 A.U.

O	0.773283	-2.359191	-0.550528
O	1.028315	2.139358	-0.222157
O	-1.057483	1.240324	-0.140252
O	2.634819	-0.094081	-1.828885
O	2.813289	-0.320235	0.427680
N	0.741728	-0.104974	-0.465144
C	0.106676	-1.355458	-0.409287
C	-1.372858	-1.428520	-0.175567
C	0.261504	1.206396	-0.262189
C	2.188897	-0.170329	-0.722664
C	-2.250038	-1.428049	-1.259241
H	-1.867246	-1.312016	-2.266318
C	-3.619414	-1.567007	-1.051597
H	-4.323356	-1.568771	-1.874084
C	-4.080187	-1.705332	0.248459
C	-3.229102	-1.723215	1.342231
H	-3.636191	-1.844341	2.338058
C	-1.861033	-1.586113	1.120569
H	-1.176198	-1.597036	1.960433
C	-1.784781	2.510796	0.129418

C	-3.233211	2.035491	0.205882
H	-3.354018	1.297615	1.000847
H	-3.536228	1.574731	-0.735791
H	-3.889468	2.884535	0.409321
C	-1.320292	3.088761	1.465078
H	-1.422098	2.341533	2.255869
H	-1.948916	3.945201	1.721518
H	-0.283564	3.418148	1.416258
C	-1.576993	3.474953	-1.036945
H	-2.219602	4.347979	-0.896964
H	-1.853616	2.994445	-1.978394
H	-0.541882	3.806775	-1.098127
C	4.298526	-0.448724	0.483550
C	4.548019	-0.612617	1.979684
H	4.181919	0.258923	2.526396
H	5.619292	-0.717629	2.165596
H	4.038708	-1.501760	2.356996
C	4.933557	0.833828	-0.050211
H	4.754398	0.951042	-1.117876
H	6.012242	0.795210	0.122181
H	4.528672	1.702819	0.472984
C	4.724216	-1.694219	-0.291512
H	4.169393	-2.566543	0.060179
H	5.790468	-1.868591	-0.125783
H	4.551645	-1.576083	-1.360147
F	-5.413907	-1.829402	0.458006

46

TS_Entry_10 SCF Done: -1552.591097 A.U.

O	0.914583	-2.338423	-0.590011
O	1.426970	2.134711	-0.195603
O	-0.707565	1.354590	-0.156662

O	2.928419	-0.166771	-1.811648
O	3.057220	-0.435558	0.443647
N	1.016464	-0.086246	-0.477147
C	0.308651	-1.297226	-0.447423
C	-1.176471	-1.282322	-0.239486
C	0.608909	1.248104	-0.260780
C	2.461433	-0.232616	-0.713518
C	-2.033668	-1.205550	-1.335473
H	-1.628695	-1.101229	-2.335256
C	-3.411980	-1.248090	-1.150424
H	-4.086446	-1.184495	-1.994574
C	-3.918766	-1.368643	0.140182
C	-3.074480	-1.467767	1.241870
H	-3.489255	-1.573379	2.236052
C	-1.697091	-1.427266	1.044718
H	-1.030408	-1.499928	1.896170
C	-1.368330	2.658978	0.125019
C	-2.841701	2.263353	0.173022
H	-3.015709	1.520198	0.952887
H	-3.156336	1.835904	-0.780394
H	-3.452955	3.143983	0.382148
C	-0.891867	3.187403	1.476765
H	-1.047513	2.434227	2.253000
H	-1.474887	4.073729	1.739259
H	0.162430	3.458327	1.448038
C	-1.089696	3.629081	-1.021338
H	-1.684024	4.534591	-0.874636
H	-1.379475	3.180952	-1.974708
H	-0.036738	3.902824	-1.062793
C	4.531310	-0.653308	0.520162
C	4.746194	-0.854404	2.017103

H	4.423249	0.028835	2.572158
H	5.806229	-1.025472	2.217776
H	4.179309	-1.717511	2.372102
C	5.249628	0.597089	0.016696
H	5.094499	0.741144	-1.051456
H	6.321279	0.491593	0.204245
H	4.888920	1.480539	0.547928
C	4.894571	-1.910168	-0.268182
H	4.282423	-2.752701	0.060263
H	5.945421	-2.151041	-0.088115
H	4.747790	-1.765583	-1.337420
Cl	-5.660072	-1.391768	0.383887

49

TS_Entry_11 SCF Done: -1430.118574 A.U.

O	1.216269	-2.299155	-0.628401
O	2.064782	2.118986	-0.170567
O	-0.121562	1.496149	-0.156107
O	3.409021	-0.271913	-1.799578
O	3.492403	-0.577249	0.453472
N	1.494529	-0.062390	-0.483180
C	0.696641	-1.213193	-0.480213
C	-0.788037	-1.090311	-0.294938
C	1.184606	1.296376	-0.252757
C	2.927821	-0.315446	-0.706723
C	-1.616190	-0.937429	-1.405808
H	-1.184193	-0.842721	-2.394901
C	-2.995192	-0.889948	-1.239849
H	-3.644160	-0.759140	-2.096588
C	-3.542084	-0.994251	0.038881
C	-2.716093	-1.171341	1.148342
H	-3.149161	-1.258362	2.136866

C	-1.337021	-1.221716	0.979502
H	-0.689052	-1.351603	1.838214
C	-0.689247	2.844250	0.129951
C	-2.187673	2.556270	0.160360
H	-2.423341	1.823309	0.933653
H	-2.522704	2.160070	-0.799588
H	-2.735650	3.477362	0.370066
C	-0.188700	3.325990	1.490166
H	-0.406838	2.580924	2.259123
H	-0.707857	4.250692	1.754192
H	0.882784	3.519381	1.473493
C	-0.329015	3.799077	-1.006233
H	-0.857583	4.744210	-0.858210
H	-0.640929	3.380171	-1.965869
H	0.741313	3.996281	-1.035306
C	4.945592	-0.908426	0.543005
C	5.127027	-1.142325	2.039462
H	4.866904	-0.243177	2.601889
H	6.168418	-1.396790	2.248982
H	4.491651	-1.963257	2.378093
C	5.762737	0.288850	0.062024
H	5.630645	0.456846	-1.005735
H	6.821019	0.098861	0.258543
H	5.465788	1.191208	0.600870
C	5.219365	-2.180263	-0.257133
H	4.541002	-2.977031	0.055033
H	6.246589	-2.503312	-0.069803
H	5.095826	-2.012589	-1.325944
C	-5.034732	-0.970498	0.216243
F	-5.647853	-0.217109	-0.726406
F	-5.396419	-0.473142	1.422373

F	-5.575539	-2.212321	0.131693

47

TS_Entry_12 SCF Done: -1185.234522 A.U.

O	0.829473	-2.353316	-0.580467
O	1.295881	2.129862	-0.200300
O	-0.826320	1.316165	-0.143526
O	2.822738	-0.162141	-1.811823
O	2.966625	-0.417387	0.444599
N	0.917262	-0.098728	-0.467398
C	0.222902	-1.312824	-0.436501
C	-1.263829	-1.317859	-0.224729
C	0.491263	1.231579	-0.255521
C	2.364551	-0.228329	-0.710410
C	-2.121670	-1.268844	-1.323325
H	-1.716588	-1.158494	-2.322117
C	-3.494572	-1.349255	-1.135598
H	-4.167667	-1.306346	-1.982893
C	-4.015131	-1.482554	0.159721
C	-3.148306	-1.552091	1.259675
H	-3.554424	-1.665818	2.257169
C	-1.776109	-1.473183	1.063062
H	-1.103485	-1.524964	1.910990
C	-1.507494	2.615491	0.124582
C	-2.974080	2.197773	0.183410
H	-3.134203	1.463954	0.975096
H	-3.286124	1.754110	-0.763487
H	-3.598273	3.071280	0.383108
C	-1.033416	3.167915	1.467224
H	-1.173529	2.422662	2.253980
H	-1.629579	4.047887	1.721129
H	0.016089	3.455732	1.430341

C	-1.247869	3.573669	-1.035850
H	-1.856138	4.471355	-0.899213
H	-1.533767	3.108540	-1.982209
H	-0.199754	3.864167	-1.084652
C	4.446347	-0.609139	0.517364
C	4.669192	-0.797789	2.014532
H	4.333389	0.083021	2.565746
H	5.732662	-0.949513	2.212285
H	4.118750	-1.668540	2.376625
C	5.139611	0.651053	0.003930
H	4.978940	0.785906	-1.064619
H	6.213526	0.565969	0.188508
H	4.765079	1.531069	0.531232
C	4.827750	-1.863601	-0.265765
H	4.233583	-2.715637	0.071108
H	5.883765	-2.084017	-0.090108
H	4.673005	-1.728012	-1.335078
C	-5.430386	-1.542432	0.359684
N	-6.573683	-1.582309	0.522581

48

TS_Entry_13 SCF Done: -1297.534183 A.U.

O	1.008163	-2.320265	-0.612583
O	1.687704	2.132334	-0.179691
O	-0.470235	1.417150	-0.138757
O	3.115058	-0.212178	-1.808828
O	3.232778	-0.501948	0.445062
N	1.206895	-0.073190	-0.474244
C	0.454811	-1.251393	-0.462882
C	-1.032911	-1.185814	-0.266251
C	0.842525	1.273494	-0.248112
C	2.648248	-0.269556	-0.710648

C	-1.873788	-1.077251	-1.374384
H	-1.452297	-0.972668	-2.366753
C	-3.251875	-1.089514	-1.203595
H	-3.927285	-0.999642	-2.043002
C	-3.764250	-1.213928	0.084071
C	-2.941717	-1.347152	1.198242
H	-3.381727	-1.452954	2.180114
C	-1.564856	-1.336137	1.014781
H	-0.904755	-1.435870	1.867947
C	-1.093121	2.744086	0.138878
C	-2.577450	2.392619	0.186395
H	-2.774785	1.661437	0.972214
H	-2.903593	1.971367	-0.766022
H	-3.162600	3.291874	0.389758
C	-0.601046	3.260971	1.489004
H	-0.780220	2.516044	2.268090
H	-1.156613	4.165604	1.748224
H	0.460956	3.499693	1.459952
C	-0.784079	3.700077	-1.011055
H	-1.350928	4.623502	-0.868180
H	-1.086616	3.257892	-1.963203
H	0.276500	3.942535	-1.052339
C	4.700947	-0.769511	0.523598
C	4.904676	-0.987377	2.019448
H	4.610664	-0.097449	2.579797
H	5.957877	-1.195223	2.221042
H	4.308756	-1.833477	2.367695
C	5.460050	0.459951	0.029376
H	5.312011	0.616169	-1.038093
H	6.527246	0.317748	0.217641
H	5.128400	1.351298	0.566267

C	5.022252	-2.032150	-0.273124
H	4.382936	-2.856542	0.049568
H	6.064378	-2.308765	-0.094209
H	4.881675	-1.875677	-1.341544
N	-5.231883	-1.198925	0.275468
O	-5.657343	-1.299175	1.419792
O	-5.934319	-1.079740	-0.720744
